# A case study of the first pregnant woman with COVID-19 in Bukavu, eastern Democratic Republic of the Congo

**DOI:** 10.1186/s40748-021-00127-5

**Published:** 2021-01-20

**Authors:** Etienne Kajibwami Birindwa, Guy Mulinganya Mulumeoderhwa, Olivier Nyakio, Guy-Quesney Mateso Mbale, Serge Zigabe Mushamuka, Jeanne Maningo Materanya, Pierrot Mulumeoderhwa Kahasha, Yvette Kujirakwinja Bisimwa, Freddy Mirindi Kampara, Jules Mongane Irenge, Isaac Barhishindi Kibalama, Pierre Kabuya Luzadi, Espoir Bwenge Malembaka, Daniel Garhalangwa-Na-Muntu Mayeri, Marius Baguma, Ghislain Bisimwa Balaluka

**Affiliations:** 1 Departement of Gynecology and Obstetrics, Hôpital Provincial Général de Référence de Bukavu (HPGRB), Bukavu, Democratic Republic of the Congo; 2 Faculty of Medicine, Université Catholique de Bukavu (UCB), Bukavu, Democratic Republic of the Congo; 3 Departement of Gynecology and Obstetrics, Panzi General Referral Hospital, Bukavu, Democratic Republic of the Congo; 4 Departement of Internal Medicine, Hôpital Provincial Général de Référence de Bukavu (HPGRB), Bukavu, Democratic Republic of the Congo; 5 Departement of Paediatrics, Hôpital Provincial Général de Référence de Bukavu (HPGRB), Bukavu, Democratic Republic of the Congo; 6 Departement of Pathology, Hôpital Provincial Général de Référence de Bukavu (HPGRB), Bukavu, Democratic Republic of the Congo; 7 Department of Anesthesiology and Intensive Care, Hôpital Provincial Général de Référence de Bukavu (HPGRB), Bukavu, Democratic Republic of the Congo; 8 Ecole régionale de santé publique (ERSP), Université Catholique de Bukavu (UCB), Bukavu, Democratic Republic of the Congo

**Keywords:** Covid-19, Inflammation, Peritoneum, Vertical transmission

## Abstract

**Introduction:**

Vertical transmission of covid-19 is possible; its risk factors are worth researching. The placental changes found in pregnant women have a definite impact on the foetus.

**Case presentation:**

We report a case of a 25-year-old woman, gravida 3, para 2 (2 alive children), with a history of two caesarean deliveries, who was infected by the SARS-CoV-2 during the last term of her pregnancy. She gave birth by caesarean at 34 weeks of gestation to a newborn baby also infected with SARS-CoV-2. The peri-operative observations noted several eruptive lesions in the pelvis, bleeding on contact. Microscopic examination of the foetal appendages revealed thrombotic vasculopathy in the placenta and in the umbilical cord vessels.

**Conclusion:**

This case is one of the first documented cases of COVID-19 in pregnancy in sub-Saharan Africa. We strongly suggest obstetricians to carefully examine the aspect of the peritoneum, viscera and foetal appendages in affected pregnant women.

## Background

In December 2019, first cases of severe acute respiratory syndrome (SARS) due to a new coronavirus (SARS-CoV-2) were reported from Wuhan in China. Soon after, the disease, subsequently named “the 2019 novel coronavirus disease” (COVID-19) and declared a pandemic by the World Health Organisation (WHO) [1], has resulted in over 18.9 million confirmed cases and more than 709,000 deaths worldwide [2].

The pregnant woman can be considered to be more at risk of severe form than the non pregnant woman [3]. Their fragile immunity and frequent comorbidities such as obesity, diabetes mellitus, arterial hypertension, or cardiovascular diseases may expose them at higher risks of developing severe forms of the disease [4] and to adverse pregnancy outcomes, especially during the third trimester [5]. COVID-19 causes pneumonia with acute respiratory distress syndrome (ARDS), which can compromise natural delivery, increase maternal morbidity, or even lead to maternal death [6]. Knowledge about coronavirus disease during pregnancy is still limited [4], and vertical transmission in utero is not yet well established [4, 5].

The risk of mother-to-child transmission of COVID-19 seems to be low [7–9]. Cases of perinatal transmission of COVID-19 have been described, but it is still unclear if this occurred via the transplacental or other routes during delivery [10]. Furthermore, COVID-19 may constitute a threat of premature delivery, intrauterine growth retardation, premature rupture of the membranes, in-utero foetal death or even a premature neonatal death during delivery or soon after [8]. Among pregnant women with SARS-CoV-2, preterm birth is reported 12.9% [11].

There are no established guidelines about the best timing nor the best mode of delivery in COVID-19 infected pregnant women to optimize foetal and maternal well-being [12]. A study on the association between mode of delivery and maternal and neonatal outcomes in COVID-19 patients in Spain has shown that caesarean section was associated with an increased risk for maternal clinical deterioration which remained significant after adjustment for confounders [13]. However, a few case-reports have shown a benefit of a caesarean section on the improvement of respiratory distress in severely affected patients [5, 12]. There have been only a few studies that have investigated the intraoperative findings in women undergoing caesarean delivery [14], or changes in the foetal appendages that may explain the risk of maternal-foetal transmission [15].

We report the first documented case of COVID-19 in a pregnant woman recorded in the province of South Kivu, in eastern Democratic Republic of the Congo (DRC) who gave birth by cesarean section to a premature newborn also infected by SARS-CoV-2. Her pelvic organs exhibited a particular inflammatory appearance, and fetal appendages revealed thrombotic vasculopathy in the placenta and in the umbilical cord vessels.

## Case presentation

A 25-year-old woman, gravida 3 para 2, at 34 weeks of gestation, with no medical history of cardiovascular nor other chronic diseases, was admitted to the labour and delivery unit of the “Hôpital Provincial Général de Référence de Bukavu” (HPGRB), in South-Kivu, for preterm labour contractions in a context of COVID-19. She had an history of 2 previous caesarean sections (the first one due to a cervical dystocia and the second indicated because of the prior caesarean), her last born wasaged 16 months. Her husband, tested negative for SARS-CoV-2, was a contact person of a COVID-19 confirmed case.

Three weeks before admission, she complained of fever, not responding to acetaminophen. Her obstetrician prescribed her antibiotics, anti-malaria, and anti-spasmodic drugs. Two weeks later, as fever persisted despite all these medications, a reverse transcriptase-polymerase chain reaction (RT-PCR) nasopharyngeal swabs was performed and confirmed she was infected by SARS-CoV-2.

She was then admitted to the provincial COVID-19 treatment center for isolation and health care. Upon arrival to the center, her body temperature was 38.7 °C. Gynecologic examination was unremarkable. All bacteriological tests, including hemocultures and cultures of urines were negative. She received antipyretics (acetaminophen), antispasmodics *trimethylphloroglucinol* and antibiotics (oral azithromycin for 5 days and intravenous ceftriaxone). Two days later, she complained of hypogastric pain, like uterine contractions of low intensity. Obstetricians of the HPGRB were contacted and recommended the administration of antispasmodics intravenously in perfusion. Despite this treatments, fever and uterine contractions persisted, so intravenous dexamethasone 12 mg daily was administered for fetal pulmonary maturation, associated with a tocolysis using nifedipine for 48 h. As the frequency, intensity and duration of contractions increased, accompanied by cervical changes (dilation, effacement, softening, and movement to a more anterior position), the patient was transferred to the labour and delivery unit of the HPGRB for an optimal care. A rapid SARS-Cov-2 antigen test was performed and found to be negative.

On admission at the HPGRB, the patient had a good general condition. Her temperature (36.5 °C) and blood pressure (120/60 mmHg) were normal. The uterine height was 29 cm, the fœtus was in cephalic presentation. On vaginal examination, the uterine cervix was softened, median, 5 mm long and had a 5 cm dilatation. Membranes were intact and the fœtal head was mobile. An obstetrical ultrasound confirmed the cephalic presentation and estimated the foetal weight at 1600 g. Foetal monitoring confirmed a foetal well-being, with a stable foetal cardiac rhythm around 140 beats per minute. Tocography showed two to three contractions per minute and an intensity of 50 to 60 mmHg. A diagnosis of intractable preterm labor in a COVID-19 patient with a history of iterative caesarean deliveries was made.

**Fig. 1:**
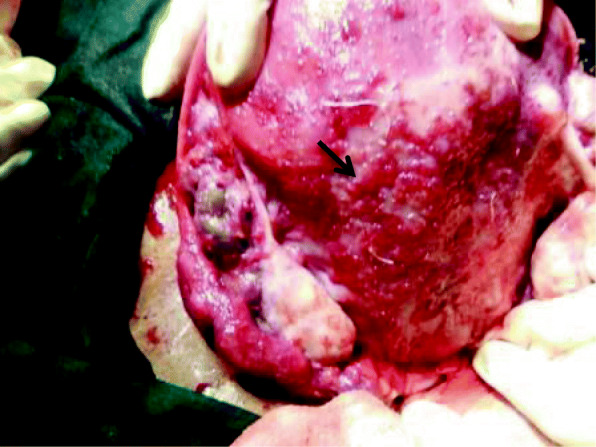
Peri-operative appearance showing several bleeding eruptive lesions on contact (  ) with the posterior surface of the uterus, on the ovaries, the left fallopian tube up to Douglas’ pouch

**Fig. 2:**
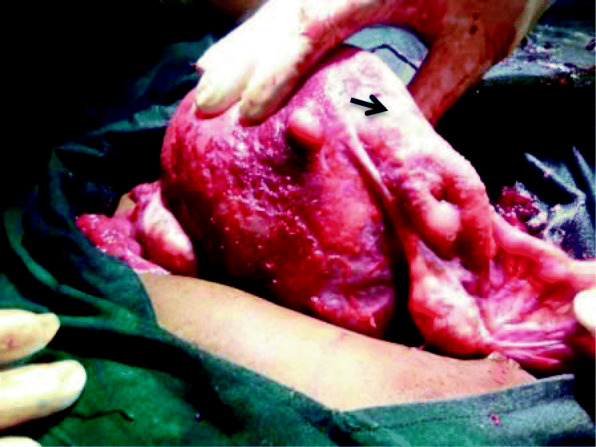
Same lesions in the right appendages of the uterus ( )

**Fig. 3:**
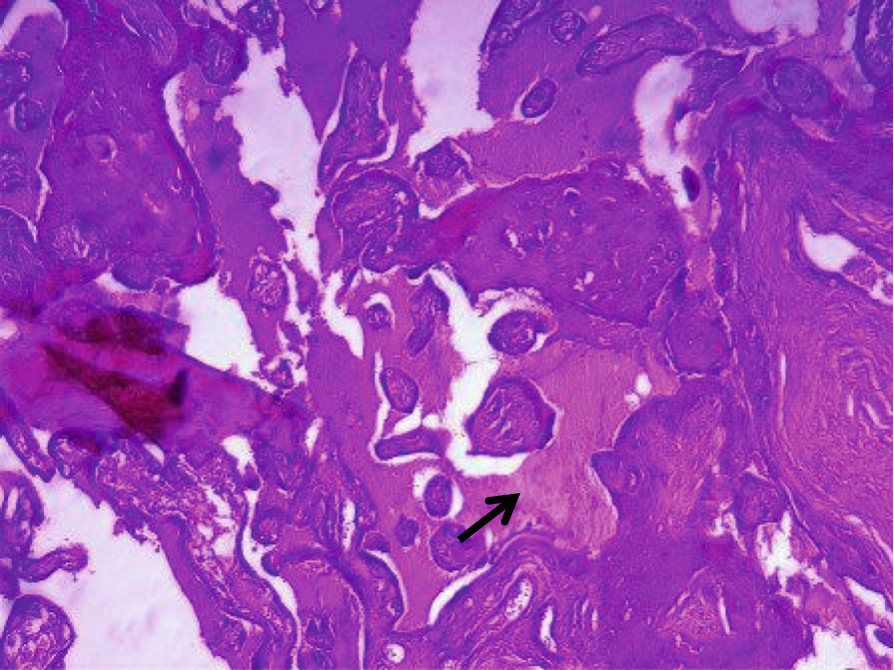
Section of the placenta; in the Hematoxylin-eosin (HE) staining we noted on sections of the placenta at 100X magnification, highly vascularized villi, with vascular lesions ( ) in the form of congestion and thrombosis visualized in certain fields and large areas of stromal hyalinization

**Fig. 4:**
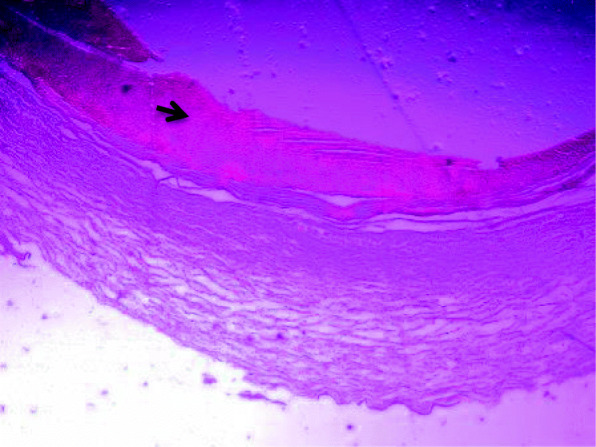
Cross section of the umbilical cord (Magnification: 40X, Hematoxylline-eosin staining). There is the presence of thrombus ( ) in the lumen of the umbilical vessel, Wharton’s jelly is without distinction, no inflammatory reaction

A classic Caesarean section with a Pfannestiel incision was performed. The peritoneal cavity and uterus were found to be very inflamed. Fetal appendages as well as the bladder were strewn with eruptive, vesicular lesions bleeding on contact **(**Figs. 1 and 2**).** The amniotic fluid was opalescent. The placenta weighed 500 g and had a clot on the maternal side on less than 20% of the surface. Anatomopathological examination subsequently revealed thrombotic vasculopathy in the placenta and in the umbilical cord vessels **(**Figs. 3 and 4**),** and a diffuse hyalinization with marked angiogenesis of the villous stroma.

About five minutes after skin incision, a female newborn weighing 1760 g was delivered with 1 and 5 min APGAR scores of 9–10. The newborn was immediately transferred to the neonatal ward for specialized neonatal treatment for an optimal care and to minimise the potential risk of infection. Gestational age was estimated at 33 weeks according to the Finnstrom score. The newborn received the usual care (drying, stimulation, vitamin K1,

Fluorescein and care of the umbilical cord). A gastric liquid was collected by gastric tube, and different swabs (especially nasopharyngeal, ear and umbilical cord), as well as blood cultures were immediately performed for bacteriological investigations and for SARS-CoV-2 RT-PCR test.

The newborn was breathing autonomously, had a good control of body temperature and blood sugar. She received a 10% glucose infusion for 48 h, and on the second day an enteral feeding by nasogastric tube was progressively introduced, using artificial milk formulas adapted to preterm babies. Prophylactic antibiotherapy (penicillin G and amikacin) was initiated, considering the risk of neonatal infections in prematurity.

On postnatal day 3, the newborn baby presented jaundice, respiratory distress and a clinical picture of ulcerative enterocolitis. Hemocultures were found negative, but SARS-CoV-2 RT-PCR was positive in oropharyngeal swab and cultures of gastric liquid isolated multiresistant Citrobacter sp. and *Enterobacter cloacae*. A phototherapy was prescribed for 3 days and previous antibiotics were replaced by meropenem and vancomycin based on the antibiogram. Despite this treatment, the newborn died on Day 5 in a picture of severe neonatal sepsis.

The postoperative follow-up of the mother was marked by a persistence of fever for 3 days, varying between 39 and 40 °C. Although haemocultures and urine cultures were sterile, antibiotic therapy was readjusted on postoperative Day 3 as for the newborn, with ceftriaxone replaced by meropenem. C-reactive protein (CRP) varied from 106.53 mg/l on admission to 186 mg/l on postoperative Day 1, falling to 21.93 mg/l on Day 5 and below 3 mg/l on Day 7. After 7 days of hospitalization, the patient’s condition was stable, with no fever nor respiratory symptoms. She was discharged from the hospital and sent back to the COVID-19 isolation Center. A control of the SARS-CoV-2 RT-PCR was negative on Day 13, she returned back home. The late postpartum up to 6 weeks was unremarkable, with no complication.

No medical staff involved in this case was subsequently found to be infected with SARS-CoV-2.

## Discussion

One year after the first cases of COVID-19, the disease continues spreading at striking speed in many countries worldwide. People get contaminated mainly through direct means (including respiratory droplets and physical contacts with carriers) or by indirect contacts with contaminated objects [16]. Vertical transmission of SARS-CoV-2 during pregnancy is another possible route of transmission [9], although further investigations are still needed to confirm this eventuality.

In previously published case reports of neonatal SARS-CoV-2 infections, it was not well established if the contamination occurred during pregnancy or after birth, especially during the delivery process [17].

Peritoneal lesions associated with SARS CoV-2 were previously suspected in patients [18–20]. Recently, the transplacental transmission of SARS-CoV-2 was reported [21, 22] and a classification of the COVID-19 infection in pregnant women, foetuses and newborns was suggested [23].

This case-report highlights a number of facts which suggest a possible intrauterine transmission of SARS-CoV-2 infection. We recognize, however, the limitations of our means of exploration which would confirm the case. Furthermore, none of the medical staff involved was subsequently found to be infected by SARS-CoV-2.

The intense inflammatory reaction of the uterus and foetal appendages suggest a direct effect of SARS-CoV-2 on placenta.

## Conclusion

This case is one of the first documented cases of COVID-19 in pregnancy in sub-Saharan Africa. The intense inflammatory reaction of the uterus and foetal appendages suggest a direct effect of SARS-CoV-2 on placenta.

We strongly suggest obstetricians to carefully examine the aspect of the peritoneum, viscera and foetal appendages in affected pregnant women.

## Data Availability

Materials and data provided in this case study are available from the corresponding author on reasonable request.

## Abbreviations

COVID-19: Coronavirus disease 2019
DRC: Democratic Republic of the Congo
HPGRB: Hôpital Provincial Général de Référence de Bukavu,
